# Olfactory marker protein contains a leucine-rich domain in the Ω-loop important for nuclear export

**DOI:** 10.1186/s13041-022-00973-0

**Published:** 2022-11-04

**Authors:** Noriyuki Nakashima, Akiko Nakashima, Kie Nakashima, Makoto Takano

**Affiliations:** 1 Department of Physiology, Kurume University School of Medicine, 67 Asahi-Machi, Kurume-Shi, Fukuoka 830-0011 Japan; 2 Department of Physiology and Cell Biology, Kobe University School of Medicine, 7-5-1 Kusunoki-Cho, Chuo-Ku, Kobe, 650-0017 Japan

**Keywords:** Olfactory marker protein (OMP), Hypothalamic neurons, Nuclear transport, Nuclear export signals (NES), Leucine-rich sequence, Bioinformatics, Multiple sequence alignment (MSA)

## Abstract

Olfactory marker protein (OMP) is a cytosolic protein expressed in mature olfactory receptor neurons (ORNs). OMP modulates cAMP signalling and regulates olfactory sensation and axonal targeting. OMP is a small soluble protein, and passive diffusion between nucleus and cytoplasm is expected. However, OMP is mostly situated in the cytosol and is only sparsely detected in the nuclei of a subset of ORNs, hypothalamic neurons and heterologously OMP-expressing cultured cells. OMP can enter the nucleus in association with transcription factors. However, how OMP is retained in the cytosol at rest is unclear. Because OMP is proposed to affect cell differentiation, it is important to understand how OMP is distributed between cytoplasm and nucleus. To elucidate the structural profile of OMP, we applied several bioinformatics methods to a multiple sequence alignment (MSA) of OMP protein sequences and ranked the evolutionarily conserved residues. In addition to the previously reported cAMP-binding domain, we identified a leucine-rich domain in the Ω-loop of OMP. We introduced mutations into the leucine-rich region and heterologously expressed the mutant OMP in HEK293T cells. Mutations into alanine increased the nuclear distribution of OMP quantified by immunocytochemistry and western blotting. Therefore, we concluded that OMP contains a leucine-rich domain important for nuclear transport.

**Supplementary Information:**

The online version contains supplementary material available at 10.1186/s13041-022-00973-0.

## Introduction

Olfactory receptor neurons (ORNs) are located in the olfactory epithelium and sense environmental chemicals for olfaction. Odourant binding to the odourant receptor in the cilia of ORNs induces intracellular cAMP signalling, which in turn opens cAMP-gated channels [1, 2]. ORNs are unique in their ability to renew throughout life. The axons of ORNs are rewired to the olfactory bulb during development to establish one-receptor–one-glomerulus neural connections that are together called the olfactory map. cAMP is crucial in both phasic olfaction and the maintenance of anatomical neural projections [1–5]. Olfactory marker protein (OMP) is a cytosolic protein that exists in cilia, dendrites, somas and axons [6–8]. OMP is related to cAMP-dependent mechanisms. OMP in the cytosol regulates cAMP kinetics [1, 2, 9]. Therefore, it is important that OMP is retained in the cytosol so that it can interact with cAMP.

OMP, at 19 kDa, is small enough to passively diffuse between the nucleus and cytoplasm [10]. Interestingly, OMP is only sparsely detected in the nuclear regions of ORNs [7]. The OMP primary structure seems to contain no intrinsic nuclear localization signals (NLSs) [11, 12]. OMP interacts with the transcription factor brain expressed and X-linked protein (Bex), which contains an NLS [7, 8, 13, 14]. Thus, Bex is believed to translocate OMP into the nucleus [7, 8]. However, the mechanisms by which OMP itself is retained in the cytosol without Bex remain unclear.

We hypothesized that OMP might contain nuclear export signals (NESs). In this study, we investigated possible nuclear transport signals in the OMP primary sequence by using several bioinformatics methods and evaluated whether the sequence could direct OMP to either the cytosol or nucleus.

## Methods

### Treatment of animals

We treated experimental animals in accordance with the Kurume University guidelines. C57BL/6N mice were purchased from SLC (Japan SLC, Inc., Shizuoka, Japan). Eight-week-old male mice were anaesthetized by intraperitoneal injection of dexmedetomidine (ZENOAQ, Fukushima, Japan), midazolam (Sandoz K.K., Tokyo, Japan) and butorphanol (Meiji Seika Pharma Co., Tokyo, Japan) at 4, 10 and 0.5 mg/kg, respectively, before rapid decapitation with a sharp blade for extraction of mRNA or proteins or before fixation by perfusion with 4% paraformaldehyde solution (PFA: FUJIFILM Wako Pure Chemical Corporation, Osaka, Japan) for immunohistochemistry.

### Reverse transcription-PCR (RT-PCR)

Anaesthetized mice were decapitated, and tissues were excised by using tweezers and scissors. The respective areas of the brain were further dissected using a scalpel. Samples were dissolved in the appropriate solution for mRNA extraction using a kit (Roche, Basel, Switzerland). cDNA was synthesized from mRNA using Superscript IV (Invitrogen, CA, USA).

### Molecular cloning of OMP

Mouse cDNA for OMP (NM_011010) cloned into the pCI mammalian expression vector was generated as previously described [2]. We used CloneAmp HiFi PCR Premix DNA polymerase (Takara Bio Inc.) to mutate the highly conserved L/T residues into alanine (OMP^A^) with the following primers: forward, 5′-GAA CTG GAC GCC CGA CGC GGC GAA CGC GAT GAC ACG CCA GGC GGC CGA CCC CGC CGC CAT CT-3′; and reverse, 5′-GTC GGG CGT CCA GTT CTG C-3′.

### Heterologous expression system

HEK293T cells (ATCC, VA, USA) were plated on coverslips and cultured in Dulbecco’s modified Eagle’s medium (D-MEM; FUJIFILM Wako Pure Chemical Corporation) supplemented with 10% foetal bovine serum (FBS: Sigma-Aldrich, MO, USA) without antibiotics at 37 °C and 5% CO_2_. HEK293T cells were transfected with 1 µg of plasmid using Lipofectamine 2000 (Thermo Fisher Scientific, MA, USA) at 50–60% confluency in D-MEM with 10% FBS. Lipofectamine 2000 reagent without the plasmid vector was used as a control. After transfection, HEK293T cells were incubated for 72 h prior to immunocytochemistry.

### Fluorescence immunocytochemistry and immunohistochemistry

For immunocytochemistry, the transfected HEK293T cells cultured on coverslips were immersion-fixed in ice-cold 4% PFA for 15 min, washed three times with PBS for more than 30 min, incubated with a primary antibody (goat anti-OMP antibody (019-22291, FUJIFILM Wako Pure Chemical Corporation) [2] at 25 °C for 6 h, washed three times with PBS and incubated with a secondary antibody (Alexa Fluor 594-conjugated anti-goat, ab150136, Abcam, Cambridge, UK) at 25 °C for 1 h. Then, the coverslips were washed with distilled water, dried, mounted on a slide (Matsunami Glass Ind., Ltd., Osaka, Japan) with VECTASHIELD antifade reagent (Vector Labs, CA, USA) and tightly sealed. For immunohistochemistry, tissues after perfusion fixation were postfixed for 10 h, cryoprotected by overnight incubation in PBS containing 30% w/v sucrose at 4 °C, mounted in OCT Embedding Compound (Sakura Finetek, Tokyo, Japan) and frontally sectioned at a 30-μm thickness using a cryostat (CM3050S, Leica Microsystems, Wetzlar, Germany) at − 20 °C. The sections were then incubated at 25 °C overnight in an appropriate blocking solution containing the following primary antibody: goat anti-OMP (019-22291, FUJIFILM Wako Pure Chemical Corporation). The samples were washed with PBS and incubated in PBS with Triton X-100 (FUJIFILM Wako Pure Chemical Corporation), 4′,6-diamidino-2-phenylindole (DAPI; diluted 1:1000; Nacalai Tesque, Kyoto, Japan) and the appropriate anti-IgG secondary antibody (diluted 1:200; Alexa Fluor 594-conjugated anti-goat) for 1.5–2 h; washed in PBS; mounted onto MAS-coated glass slides (Matsunami Glass Ind., Ltd.); coverslipped using VECTASHIELD antifade reagent (Vector Labs, CA, USA); and tightly sealed. The above procedures were conducted at 25 °C. Fluorescence signals were detected using a fluorescence microscope (BX50, Olympus, Tokyo Japan), imaged with a digital camera (DP72, Olympus) and analysed with cellSens image analysis software (Olympus). The images were taken by a water-immersion objective (LUMPlanFl 60x/0.90 w: Olympus).

### Cell fractionation and western blotting

The subcellular components of the hypothalamus from 4-week-old male mice and HEK293T cells transfected with OMP^WT^ or OMP^A^ were isolated by using a nuclear/cytosol fractionation kit (BioVision Incorporated, MA, USA). After isolation, the solutions were mixed with Laemmli sample buffer (Bio-Rad Laboratories, CA, USA) at 25 °C, supplemented with 10% 2-mercaptoethanol (Sigma-Aldrich, MO, USA), heated at 95 °C for 5 min and used for subsequent western blotting. Then, 5-μL volumes of the samples were electrophoresed using a precast 12% Mini-PROTEAN TGX gel (Bio-Rad Laboratories) in a Mini-PROTEAN Tetra cell (Bio-Rad Laboratories) according to the manufacturer’s instructions. Then, the proteins were transferred to PVDF membranes (Midi Format 0.2 µm PVDF Cat. #1704157, Bio-Rad Laboratories, CA, USA) using a Trans-Blot Turbo Transfer System (Bio-Rad Laboratories, CA, USA) for 7 min at 25 V. The PVDF membranes were incubated with primary antibodies against OMP (goat; 019-22291, FUJIFILM Wako Pure Chemical Corporation) [15], α-tubulin (mouse, 66031-1-IG, Proteintech Group, Inc., IL, USA) [16], Lamin-A/C (rabbit, 10298-1-AP, Proteintech Group) [17] or glyceraldehyde-3-phosphate dehydrogenase (mouse, GAPDH; 3H12, MBL Co. Ltd, Tokyo, Japan) [18] (all 1:1000) diluted with Can Get Signal Immunoreaction Enhancer Solution 1 (NKB-101, Toyobo, Osaka, Japan) overnight at 4 °C and washed with 0.1% Tween in Tris-buffered saline (TBST: 10 mM Tris; 150 mM NaCl; pH 7.6) 3 times for 10 min each. The membranes were incubated with an anti-rabbit secondary antibody conjugated with horseradish peroxidase (HRP; 7074P2, Cell Signaling Technology), an anti-goat HRP-conjugated secondary antibody (ab6885, Abcam) or an anti-mouse HRP-conjugated secondary antibody (ab6823, Abcam, Cambridge, UK) all at a 1:2000 dilution in Can Get Signal Immunoreaction Enhancer Solution 2 (NKB-301, Toyobo) for 1.5 h at 25 °C and then washed with TBST 3 times for 10 min each. Then, the membranes were washed carefully. Immunoreactivity was detected using ECL Prime western blot detection reagent (RPN2232, Cytiva, NJ, USA). The images were captured using an Amersham Imager 600 (GE Healthcare Biosciences, NJ, USA). To detect several immunoreactivities, the membranes were stripped of the first set of primary and secondary antibodies by using WB Stripping Solution (05364-55, Nacalai Tesque) and sequentially reprobed with the next set of primary and secondary antibodies.

### Multiple sequence alignment (MSA)

To draw a representative MSA of OMP in Fig. 2a, we obtained the aligned sequences of OMP with STRAP [19] by inputting the following list of accession numbers: AAA20485.1 (*Homo sapiens*), XP_004051907.1 (*Gorilla gorilla gorilla*), P08523.2 (*Rattus norvegicus*), XP_006004323.1 (*Latimeria chalumnae*), XP_004938999.1, (*Gallus gallus*), XP_015266020.1 (*Gekko japonicus*), AAI70182.1 (*Xenopus laevis*), and TWW75162.1 (*Takifugu flavidus*). To further construct the MSA of all available species, we collected information on the protein orthologues by consulting the NCBI Constraint-based Multiple Alignment Tool (COBALT [20]). The MSA was obtained in FASTA format and transformed into a spreadsheet by using original programmes (Additional files 2 and 3).

### Structural model simulation

We obtained the crystal structures of OMP [21] (1zri) from the Protein Data Bank (PDB). We used PyMOL (The PyMOL Molecular Graphics System, Version 2.0 Schrödinger, LLC., NY, USA) and AutoDock Vina [22] (The Scripps Research Institute, CA, USA) for structural modelling. For docking simulations, the protein data were further modified by adding polar hydrogen atoms and rendered into an analysis grid using AutoDock Tools (version 1.5.6) [22]. Selenomethionine (MSE in the PDB data) was not edited.

### Hydrophobicity

Hydrophobicity was calculated by using an online prediction site (https://www.expasy.org/).

### Nuclear transport signal prediction

In searching an NLS or NES, we consulted NLStradamus [23], NLS mapper [24], NetNES [25] and NLSdb [26]. Neither predicted possible NLSs nor NESs in OMP.

### Bioinformatics metrics on OMP

We used three metrics, a taxonomy-based metric (LIST-S2) [27], the first-order moment (probability in the form of information entropy) and the second-order moment, as defined below. The LIST-S2 scores for OMP were obtained online [27]. The information entropy at the *i*th partition with amino acid diversity (Di) and its emergence probability (P_Di_) was calculated as follows [28]:

1 $$\text{Information}\; \text{Entropy}={\sum }_{Di=1}^{20}{P}_{Di}{log}_{e}{P}_{Di}$$

Note that for the information entropy in this report, the negative inverse was used for visual comparison of the spectral forms with other metrics.

To formulate the second-order moment, we reconsidered the probability density function for the appearance of diverse amino acids at a certain *i*th residue within MSA. In an orthologue set consisting of the total number of species, N_sp_, a given amino acid partition of the protein presented amino acid *diversity* (D) ranging from 1 to 20. To evaluate the variation with respect to the most restrained residue, we considered the *difference* value (d) from the most abundant residue by subtracting 1 from the *diversity*, namely, *d* = *D* − 1 (d; 0 ≤ d ≤ 19). Next, the moment-generating function (M_d_(t)) for random discrete variables of amino acids, where d is a mutation diversity d and an arbitrary parameter t, was defined as follows by using the Taylor series expansion:

2 $${\text{M}}_{d}\left(\text{t}\right)\equiv 1 + \frac{E\left[d\right]}{1!}t+\frac{E\left[{d}^{2}\right]}{2!}{t}^{2}+\cdots$$

where *E*[*d*] is an expected value and *E*[*d*^*2*^] is the secondary moment related to variance. By using d, Nsp and the respective species abundance N_i_(d), *E*[*d*^*2*^] was formulated as follows:

3 $$E\left[{d}^{2}\right]= \frac{1}{{N}_{sp}}[1+{\sum }_{d=0}^{19}{d}^{2} {N}_{i}\left(d\right)]$$

Note that the initial term (= 1) in the brackets of Eq. 3 is a score arbitrarily endowed as a value of existence for the *i*th residue within a primary sequence regardless of N_sp_ or N_i_(d). In Eq. 3, the terms in the brackets are defined as φ_i_:

4 $${\varphi}_{\text{i}}\equiv 1+{\sum }_{d=0}^{19}{d}^{2} {N}_{i}(d)$$

Therefore, the secondary moment *E*[*d*] was equivalent to the φ_i_ value divided by N_sp_; namely, *E*[*d*] = φ_i_/N_sp_. However, *E*[*d*] could be overestimated if the sample species were limited at a certain partition. Instead, we further normalized the secondary moment *E*[*d*^*2*^] by dividing the value by N_sp_ to yield Φ_i_, as follows:

5 $${\Phi }_{\text{i}}=\frac{E\left[{d}^{2}\right]}{{N}_{sp}}=\frac{{\varphi}_{\text{i}}}{{\left({N}_{sp}\right)}^{2}}$$

To elucidate the conservation profile, we transformed Φ_i_ into a negative logarithmic form, as follows:

6 $${\kappa }_{\text{i}}=-{\ln}{\Phi }_{\text{i}}$$

In mathematical analogy to the kinetic *moment of inertia* (I) of a rotating object with mass (M_r_) around radius (r) as I ≡ r^2^M_r_, the value Φ_i_ was formulated in the same dimensions as the kinetic moment. Therefore, the value κ_i_ was named the phylogenetic kinetic value (PKV).

## Results

### OMP may be actively localized in the cytoplasm

**Fig. 1:**
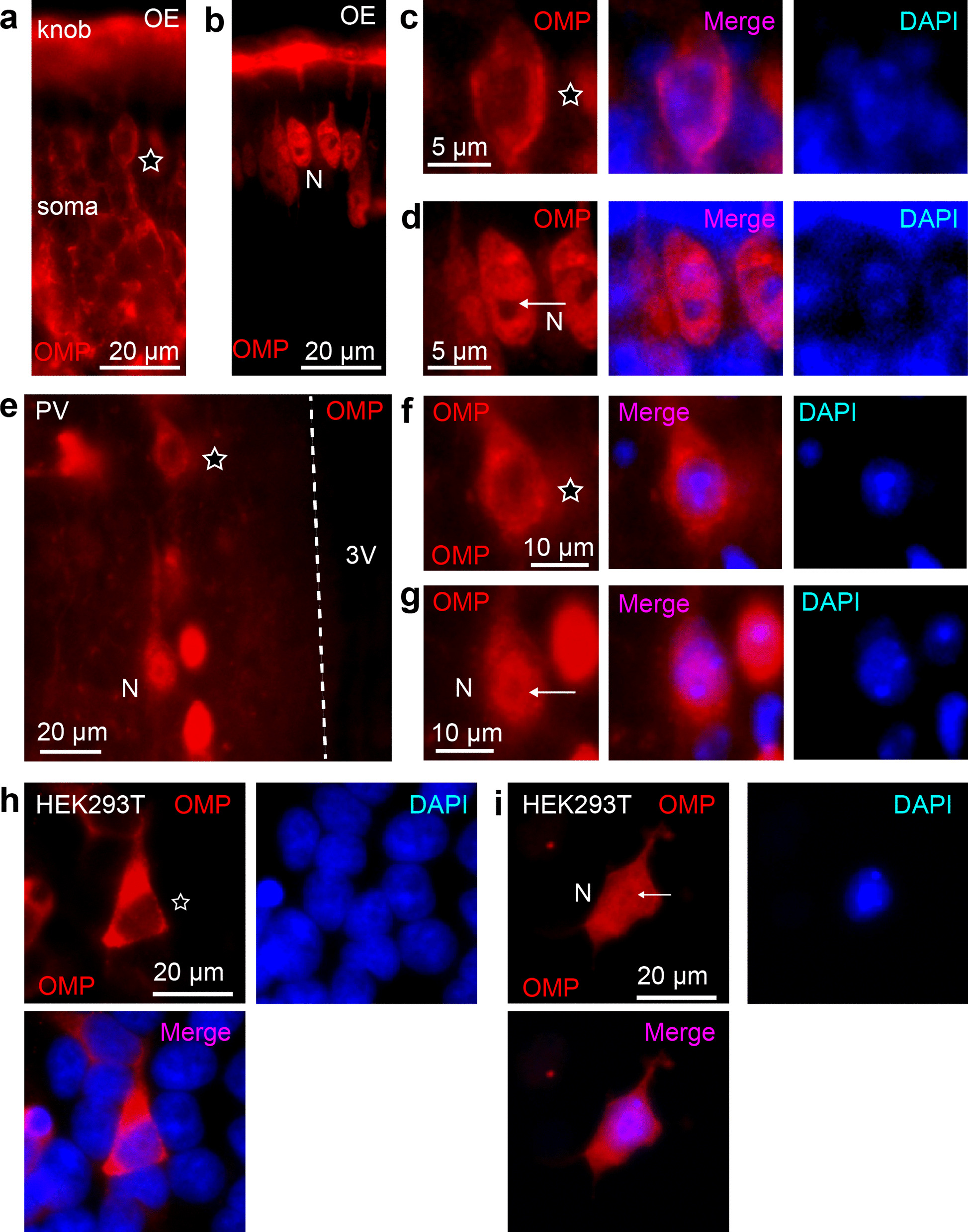
OMP occasionally enters the nucleus in neurons and cultured cells. **a**, **b** OMP-IR in the mouse olfactory epithelium (OE). OMP-IR was detected in the cytosol from dendritic knob to somas. OMP-IR was usually not detected in the nuclei of ORNs (**a**), but some ORNs exhibited OMP-IR inside nuclei. **c**, **d** Closed views of ORNs with OMP (**c**) exclusively in the cytosol or occasionally (**d**) in the nucleus. Note that OMP-IR even in the nucleus was excluded from dense nuclear foci in diamidino-2-phenylindole (DAPI) staining (white arrow). **e** OMP-IR was detected in neurons in the paraventricular nucleus (PV) of the hypothalamus. **f**, **g** Close views of neurons in the paraventricular neurons with OMP-IR **f** exclusively in the cytosol or **g** in both the cytosol and the nucleus. 3V, third ventricle. **h**, **i** OMP-IR in HEK293T cells. OMP-IR was detected either **h** exclusively in the cytosol or **i** in both the cytosol and the nucleus. The cells with OMP-IR in the cytosol or in the nucleus are labelled with an open star or N, respectively, in **a**–**i**

OMP immunoreactivity (OMP-IR) was normally detected in ORNs and hypothalamic neurons (Fig. 1a–g; Additional file 4: Fig. S1) [13]. Close observation revealed that OMP-IR was frequently detected only in the cytoplasm; some populations of cells exhibited OMP-IR both in the cytoplasm and nucleus (Fig. 1d, g), as previously reported [7, 13]. The structural voids of OMP-IR within the nucleus were dense nuclear foci in DAPI staining. When heterologously expressed in HEK293T cells, OMP-IR was detected only in the cytoplasm or both in the cytoplasm and nucleus (Fig. 1h, i) [7]. Thus, OMP may be actively localized in the cytoplasm on some occasions. This observation let us hypothesize that OMP might contain any recognition motifs for nuclear exports.

### OMP contains a leucine-rich region in the Ω-shaped loop

**Fig. 2:**
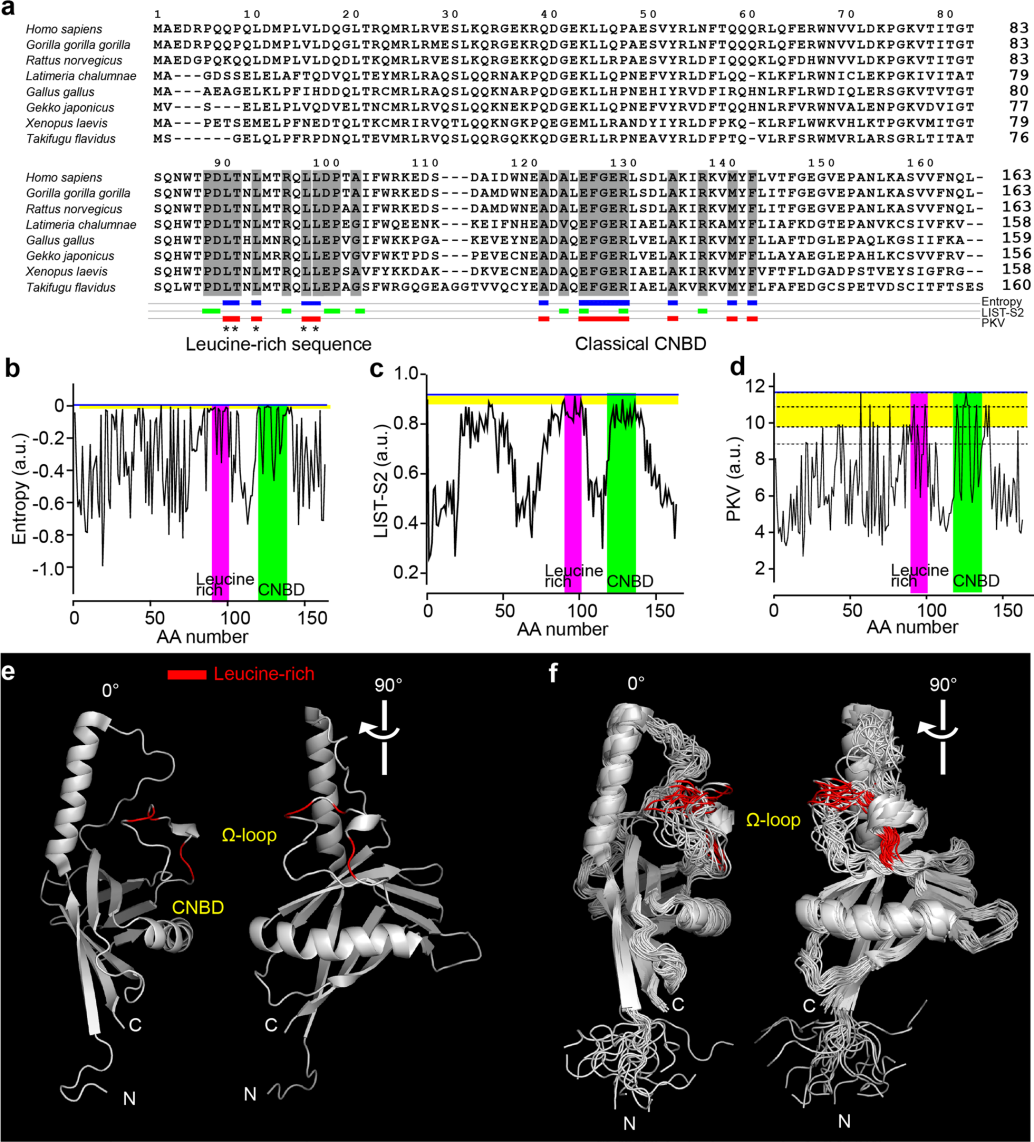
OMP contains a leucine-rich sequence in the Ω-loop. **a** Spectral views of the MSA of OMP proteins from various vertebrate phylogenies. The amino acid number was expanded to the comprehensive serial number. Highly scored residues by **b**, **c** around the leucine-rich sequence and cyclic nucleotide-binding domain (CNBD) are highlighted. **B**–**d** Spectral views for the **b** entropy, **c** LIST-S2 metric and **d** PKV. In **d**, the dotted lines were added for visibility of the similarly scored distant residues. **e** Crystal structure of OMP. A highly conserved leucine-rich sequence region was detected in the Ω-loop of OMP. CNBD, cyclic nucleotide-binding domain. **f** Overlay of different conformations of OMP depicting that the Ω-loop is flexible

We applied bioinformatics to elucidate the evolutionarily conserved residues within the primary structure of OMP (Fig. 2a). The conventional information entropy [28] and the recently released taxon-related LIST-S2 [27] elucidated the cyclic nucleotide-binding domain (CNBD) at approximately sites 120–130 (Fig. 2a–c) [2], but the entropy profile results were rather spiky (Fig. 2b). LIST-S2 predicted the large three clusters, but the peaks were slightly different from the entropy profile (Fig. 2a, c; Additional file 1). Thus, we aimed to construct another metric that utilized the interspecies variance-related value in the mutation frequency at each residue across the MSA. This value was calculated as the secondary moment of frequency distribution and should describe an aspect of the evolutionary mutation kinetics among phylogenies by taking an analogous form of the kinetic moment (see “Methods”). Thus, the value was named the phylogenetic kinetic value (PKV). The PKV distinctly classified the distant residue clusters with similar scores (Fig. 2d). In addition to CNBD, PKV highlighted the residues with the highest scores spanning 90–100, where the high-scored residues were leucine (Fig. 2a, d). These peaks were the same as the entropy peaks (Fig. 2a). The leucine-rich domain was in an Ω-loop of OMP [29], which was exteriorly exposed and flexible to take multiple conformations (Fig. 2e, f). This sequence signature resembles those of leucine-rich NESs [25].

### Mutations in the leucine-rich sequence alter the nuclear transport of OMP

**Fig. 3:**
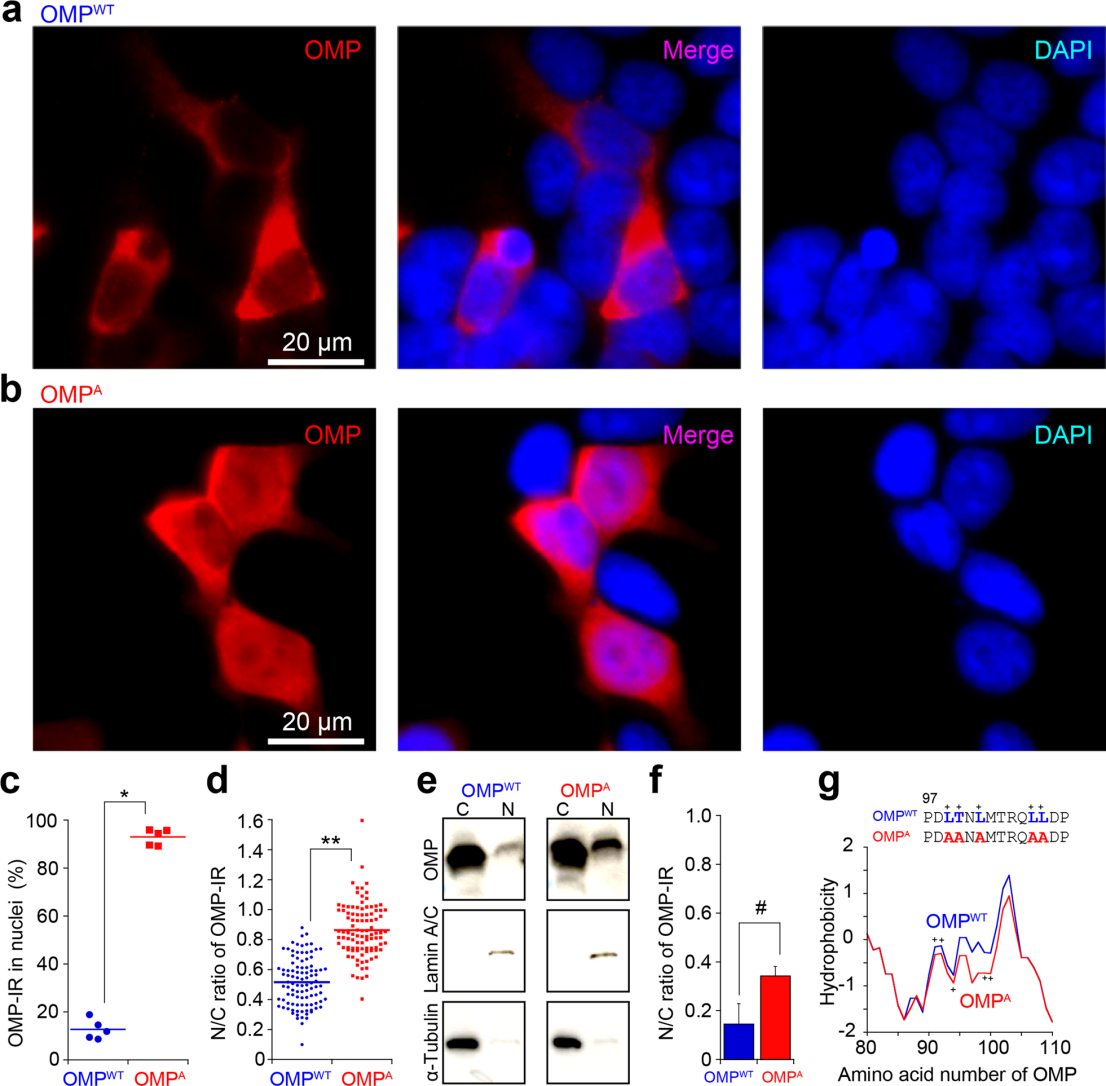
Mutations in the leucine-rich sequence alter the nuclear export efficacy of OMP. **a**, **b** Representative pictures of OMP-IR in HEK293T cells expressing **a** OMP^WT^ or **b** OMP^A^. **c** Quantification of the cell numbers (%) showing nuclear OMP-IR with apparent signal voids surrounding dense nuclear foci among all OMP-IR cells transfected with OMP^WT^ (filled blue circles) and OMP^A^ (filled red squares). n = 5 experiments each. One-tailed Student’s T test: OMP^WT^ vs. OMP^A^, *P = 7.36 × 10^–10^. **d** The OMP-IR ratio of the nucleus/cytoplasm (N/C ratio). n = 100 cells for OMP^WT^ and 102 cells for OMP^A^. One-tailed Student’s T test: OMP^WT^ vs. OMP^A^, **P = 6.97 × 10^–34^. **e** OMP-IR from cytoplasmic (C) and nuclear (N) fractions extracted from HEK293T cells expressing OMP^WT^ or OMP^A^. **f** N/C ratio for the cell-fractional OMP-IR detected by western blotting in **e**. One-tailed Student’s T test: OMP^WT^ vs. OMP^A^, ^#^P = 0.0089. **g** Hydrophobicity of OMP^WT^ (blue) and OMP^A^ (red) around the Ω-loop. +, mutated residues

We hypothesized that this leucine-rich domain should be important for the nuclear transport of OMP and mutated the highly conserved leucine/threonine residues to alanine (L91A/T92A/L94A/L99A/L100A designated OMP^A^). When wild-type OMP (OMP^WT^) was expressed in HEK293T cells, we confirmed that OMP^WT^-IR in the nucleus was detected in a small portion of the cultured cells (Fig. 3a). In contrast, compared to OMP^WT^-IR, OMP^A^-IR was significantly more frequently detected in the nucleus surrounding the dense nuclear foci in DAPI staining, with residual immunoreactivity in the cytosol (Fig. 3b, c). OMP^A^-IR showed a significantly higher nuclear/cytoplasmic signal intensity ratio (N/C ratio) than OMP^WT^-IR in the cells (Fig. 3d). The nuclear and cytoplasmic fractions from HEK293T cells expressing OMP^WT^ or OMP^A^ were quantified, which also resulted in a higher N/C ratio in OMP^A^-IR cells than in OMP^WT^-IR cells (Fig. 3e, f). The mutations affected the hydrophobicity around the Ω-loop (Fig. 3g), which may change the interaction with the transport cargo proteins [30]. In conclusion, these results indicate that the leucine-rich region is important for the nuclear export of OMP.

## Discussion

In the present study, we identified a leucine-rich sequence associated with the nuclear export process of OMP. The nuclear translocation of proteins larger than approximately 50 kDa may require transport receptors [30]. Because OMP weighs only 19 kDa, the passive diffusion of OMP should be sufficient to distribute to the cytoplasm at least [10]. Indeed, OMP was detected mostly in the cytoplasm in a subset of cells (Fig. 1) [7], and the active export of OMP out of the nucleus could be physiologically important under certain conditions.

Considering that OMP^A^-IR in the nuclei was observed only in the cells with visible dense nuclear foci in DAPI staining, it is possible that OMP^A^-IR was detected in a certain cell cycle phase or that the cellular conditions were disturbed [7]. Notably, OMP has a clam-shell-like flexible structure [29], and OMP has different turnover rates depending on the multimeric forms [7, 31], although an exact model for the nuclear localization of OMP has not been proposed. As one of the interaction candidates, Bex1 is proposed to translocate OMP into the nucleus by directly binding to OMP near the Ω-loop [29]. These findings let us speculate that OMP with an NES is retained in the cytosol at rest and that some binding proteins, such as Bex1, may cover the NES to translocate OMP or possibly allow a small complex with OMP to passively diffuse into the nucleus. Bex proteins are transcription factors and intrinsically disordered proteins [14] and can regulate cell proliferation and survival [7, 32, 33]. The nuclear OMP translocated by Bex is suspected to control the gene expression of cells for cell maturation and neural projection [1–4, 34]. The Ω-loop underlies protein conformation and interactions with other molecules and the subsequent physiological functions [35, 36]. Notably, OMP^WT^-IR was mostly excluded from dense nuclear foci in DAPI staining but was slightly detected in the nucleus at rest (Figs. 1g, h; 3a) [7]. This could be partly because the leucine alignment in the Ω-loop of OMP is similar to but slightly different from that of the conventional NES [25]. The detailed actions of the leucine-rich sequence in the nuclear translocation of OMP as well as the partner molecules, including Bex1, need clarification.

To identify the vital residues with unknown functions in OMP, we utilized a secondary moment-based metric defined as PKV. With the aid of bioinformatics, putative functional residues might be highlighted [27, 28]; the completely or mostly conserved residues are especially detectable. However, for many proteins, vital residues and their actions remain unknown mostly because the effects of mutations may be deleterious [27] and eliminated from genetic pools or subtle and compensated for by other molecules. In such cases, phenotype-based investigations, including classical forward genetics investigations [37–39], might be less suitable for quantitative evaluation. In addition, proteins with pleiotropic functions may possess multiple sites of interactions [2, 7, 40] and have no characterized paralogues, such as OMP [2, 41]. Thus, the de novo identification of vital residues with moderate conservation profiles and the reverse-genetics study should be important to assess the molecular action of the protein. In this study, the secondary moment can be recapitulated as a driving force that shapes the current mutation frequency within the MSA and could be used to speculate on the putative past mutation kinetics rather than the diversity itself. In fact, PKV effectively highlighted multiple clusters, including cAMP-binding [2] and leucine-rich motifs. Notably, some other high-scored cohort residues with unknown actions in the N-terminus were also highlighted by PKV in Fig. 2c; this domain has been unidentified. Although the evolutionarily conserved residues were clustered by PKV, the molecular actions of these regions should be predicted according to the characteristics of the residues to some extent and experimentally verified through reverse-genetics manipulations and biophysical experiments [2, 42]. As an option to start with, PKV could be helpful in combination with other metrics [27, 28] and de novo approaches to study important residues, such as directed evolution [43, 44] and large-scale mutagenesis [45].

## Supplementary Information

**Additional file 1:** The raw data presented in this study.

**Additional file 2:** The raw MSA of OMP in FASTA format downloaded from COBALT.

**Additional file 3:** The Macrocodes to convert the MSA in a FASTA format into an Excel format.

**Additional file 4: Figure S1.** OMP was expressed in both cytoplasmic and nuclear fractions of hypothalamic tissue.

## Data Availability

All data and codes generated or analysed during this study are included in this published article and Additional files.

## Abbreviations

3V: Third ventricle
BEX: Brain expressed and X-linked protein
CNBD: Cyclic nucleotide-binding domain
GAPDH: Glyceraldehyde-3-phosphate dehydrogenase
IR: Immunoreactivity
MSA: Multiple sequence alignment
N/C ratio: Nuclear/cytoplasmic signal intensity ratio
NES: Nuclear export signal
NLS: Nuclear localization signal
OE: Olfactory epithelium
OMP: Olfactory marker protein
ORN: Olfactory receptor neuron
PFA: Paraformaldehyde solution
PKV: Phylogenetic kinetic value
WT: Wild-type
