# Supplementary material for: Olfactory marker protein contains a leucine-rich domain in the Ω-loop important for nuclear export
Source: Mol Brain. 2022 Nov 4;15:89. doi: 10.1186/s13041-022-00973-0 (PMC9636679; doi:10.1186/s13041-022-00973-0)
Supplement: Supplementary file 4 — Additional file 4: Figure S1. OMP was expressed in both cytoplasmic and nuclear fractions of hypothalamic tissue. [file 13041_2022_973_MOESM4_ESM.docx]

**Additional file for**

**Title: Olfactory marker protein contains a leucine-rich domain in the Ω-loop important for nuclear export**

**Authors:**

Noriyuki Nakashima^1^*^#^, Akiko Nakashima^1^*^#^, Kie Nakashima^2^*^#^, Makoto Takano^1^

**Affiliations:**

^1^ Department of Physiology, Kurume University School of Medicine, 67 Asahi-machi, Kurume-shi, Fukuoka, 830-0011, Japan.

^2^ Department of Physiology and Cell Biology, Kobe University School of Medicine, 7-5-1 Kusunoki-cho, Chuo-ku, Kobe, 650-0017, Japan.

**Contact information:**

Noriyuki Nakashima: [nakashima_noriyuki@med.kurume-u.ac.jp](mailto:nakashima_noriyuki@med.kurume-u.ac.jp)

Akiko Nakashima: [nakashima_akiko@med.kurume-u.ac.jp](mailto:nakashima_akiko@med.kurume-u.ac.jp)

Kie Nakashima: [nakasima@med.kobe-u.ac.jp](mailto:nakasima@med.kobe-u.ac.jp)

Makoto Takano: [takanom@med.kurume-u.ac.jp](mailto:takanom@med.kurume-u.ac.jp)

* **Correspondences:**

Noriyuki Nakashima: [nakashima_noriyuki@med.kurume-u.ac.jp](mailto:nakashima_noriyuki@med.kurume-u.ac.jp)

Akiko Nakashima: [nakashima_akiko@med.kurume-u.ac.jp](mailto:nakashima_akiko@med.kurume-u.ac.jp)

Kie Nakashima: [nakasima@med.kobe-u.ac.jp](mailto:nakasima@med.kobe-u.ac.jp)

Phone: +81-942-31-7543

Fax: +81-942-31-7728

**# Equal contributions**

**Figure S1**

**
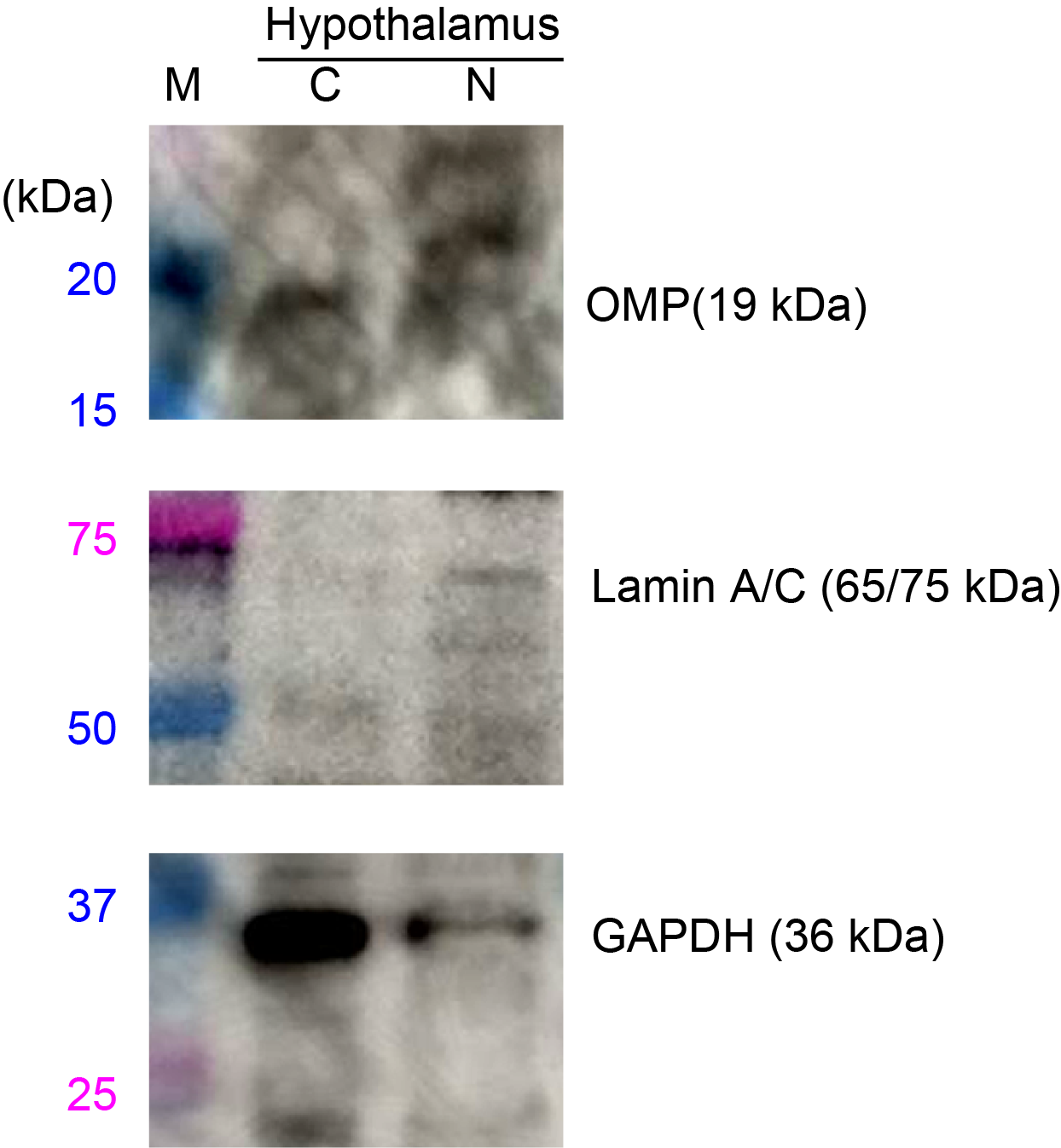
**

**Legend for Figure S1**

**OMP was expressed in both cytoplasmic and nuclear fractions of hypothalamic tissue.** Olfactory marker protein immunoreactivity (OMP-IR) was detected weakly in both the cytoplasmic and nuclear fractions. In the nuclear fraction, OMP-IR was also detected in the region for higher molecular weights**.** In hypothalamic extract, glyceraldehyde-3-phosphate dehydrogenase-IR (GAPDH-IR) was used to identify the cytoplasmic fraction. M, marker; C, cytoplasmic fraction; N, nuclear fraction. Exposure: 60 s for OMP, 5 s for Lamin A/C and 30 s for GAPDH.
